# Laser Processing and Multi-Energy Field Manufacturing of High-Performance Materials

**DOI:** 10.3390/ma16175991

**Published:** 2023-08-31

**Authors:** Xiaoxiao Chen, Yaou Zhang, Anhai Li

**Affiliations:** 1 Research Centre for Laser Extreme Manufacturing, Ningbo Institute of Materials Technology and Engineering, Chinese Academy of Sciences, Ningbo 315201, China; 2 Zhejiang Key Laboratory of Aero Engine Extreme Manufacturing Technology, Ningbo 315201, China; 3 University of Chinese Academy of Sciences, Beijing 100049, China; 4 School of Mechanical Engineering, Shanghai Jiao Tong University, Shanghai 200240, China; yaou_zhang@sjtu.edu.cn; 5 School of Mechanical Engineering, Shandong University, Jinan 250061, China; anhaili@sdu.edu.cn

The laser is one of the major inventions of the 20th century, along with atomic energy, the computer and semiconductors. Laser processing technology is non-contact, which makes it suitable for the processing and manufacturing of various materials without using cutting forces. During the machining process, the macro-/micro-processing of mechanical motions and the high-speed scanning of galvanometers can be realized. Compared with traditional processing, this has significant advantages in some aspects of the field.

Today, with the development of materials science and technology, there is an endless stream of various new emerging materials. The technical demand for material applications is constantly increasing, and advanced materials have been widely used in various fields. At the same time, composite processing technology is also gradually developing. The composite manufacturing of multiple energy fields can benefit from the advantages of various single energies. After the optimization of various energy field combinations, the high-performance processing of materials can be achieved.

This Special Issue summarizes recent advances in the fields of laser processing and multi-energy field composite manufacturing. The ten articles published in this Special Issue cover a variety of topics, including laser cladding, laser coating, laser-based directed energy deposition, laser cutting, laser grooving, laser drilling, electric discharge machining, ultrasonic burnishing, and ultrasonic-vibration-assisted pressing process methods. The effects of lasers, vibrations, electricity and other energies on the properties and processing techniques of various high-performance materials, such as medium entropy alloys, refractory high entropy alloys, high-temperature alloys Inconel 718, carbon fiber reinforced composites, ceramic based composites, diamond materials, aluminum alloys, and hard alloys, have been fully analyzed and discussed.

This Special Issue aims to showcase the latest achievements in the fields of laser processing and multi-energy field composite manufacturing, solicit the most important discoveries, highlight the challenges of processing mechanisms, theories and technologies, and provide an outlook on future directions. The article publication status of this Special Issue is as follows.

Laser cladding is an effective method for the surface modification of matrix materials. The preparation of functional coatings on metal substrates is an effective method to enhance the surfaces of steel structures, with good serviceability in applications for engineering parts. However, there are few studies on the UB strengthening of MEA laser-cladding coating. Shen et al. [1] investigated the surface properties of two sorts of medium-entropy alloy (MEA) coatings (CoCrNi coating and FeCoNiCr coating) prepared using laser cladding. After cladding, the two prepared coatings were strengthened with ultrasonic burnishing (UB) treatment. Cladding coating samples were comparatively tested before and after being UB-treated in order to investigate the process effects of UB. When compared with corresponding untreated coating samples, the roughness values of the two sorts of UB-treated samples were decreased by 88.7% and 87.6%, the porosities were decreased by 63.8% and 73.4%, and the micro-hardness values were increased by 41.7% and 32.7%, respectively. Furthermore, the two sorts of UB-treated coating samples exhibited better mechanical properties and wear resistance than corresponding untreated samples.

In addition, Xu et al. [2] investigated the effects of the Cr content on the microstructure and properties of the WVTaTiCr_x_ (x = 0, 0.25, 0.5, 0.75, 1) coating. The trends in the coating’s microstructure, hardness, high-temperature oxidation resistance, and corrosion resistance were analyzed by varying the Cr content. As a result, with the increase in Cr, the coating grains were more refined. The coatings were mainly composed of the BCC solid-solution phase, which promotes the precipitation of the Laves phase with the increase in Cr. The addition of Cr greatly improved the hardness, high-temperature oxidation resistance and corrosion resistance of the coating. The WVTaTiCr (Cr_1_) exhibited superior mechanical properties, especially in terms of its exceptional hardness, high-temperature oxidation resistance and outstanding corrosion resistance. The average hardness of the WVTaTiCr alloy coating reached 627.36 HV. After 50 h of high-temperature oxidation, the oxide weight of WVTaTiCr increased by 5.12 mg/cm^2^, and the oxidation rate was 0.1 mg/(cm^2^·h). In the 3.5 wt% NaCl solution, the corrosion potential of WVTaTiCr was −0.3198 V and the corrosion rate was 0.161 mm/a.

The profile of the laser beam plays a significant role in determining the heat input on the deposition surface, further affecting the molten pool dynamics during laser-based directed energy deposition. However, there has been little research on the laser–powder interaction and corresponding heat transport of powders under the laser intensity input with super-Gaussian distribution. Chen et al. [3] proposed an improved thermal-fluid model including a laser–powder interaction model and a metal deposition model to explore the thermal-fluid transport and solidification characteristics under two types of laser beams (Gaussian and super-Gaussian) during the single-track L-DED process of Inconel 718. The deposition surface of the molten pool was calculated using the Arbitrary Lagrangian Eulerian moving mesh approach. Several dimensionless numbers were used to explain the underlying physical phenomena under different laser beams. Moreover, the solidification parameters were calculated using the thermal history at the solidification front. It was found that the peak temperature and liquid velocity in the molten pool under the SGB case were lower compared with those for the GB case. Dimensionless numbers analysis indicated that the fluid flow played a more pronounced role in heat transfer compared to conduction, especially in the GB case. The cooling rate was higher for the SGB case, indicating that the grain size could be finer compared with that for the GB case. Finally, the reliability of the numerical simulation was verified by comparing the computed and experimental clad geometry. The work provides a theoretical basis for understanding the thermal behavior and solidification characteristics under different laser input profiles during directed energy deposition.

Primary dendrite arm spacing (PDAS) is a crucial microstructural feature in nickel-based superalloys produced by laser cladding. Currently, most multi-scale simulations of laser cladding use the finite element method with birth-death elements. However, these approaches do not account for changes in the free surface or fluid flow in the molten pool and the latent heat of melting. Jin et al. [4] proposed a multi-scale model that integrates a 3D transient heat and mass transfer model with a quantitative phase-field model to simulate the dendritic growth behavior in the molten pool for laser cladding Inconel 718. The values of the temperature gradient (G) and solidification rate (R) at the S/L interface of the molten pool under different process conditions were obtained via multi-scale simulation and used as the inputs for the quantitative phase field model. The influence of process parameters on the microstructure morphology in the deposition layer was analyzed. The result shows that the dendrite morphology was in good agreement with the experimental result under varying laser power (P) and scanning velocity (V). PDAS was found to be more sensitive to changes in the laser scanning velocity, and as the scanning velocity decreased from 12 mm/s to 4 mm/s, the PDAS increased by 197% when the laser power was 1500 W. Furthermore, smaller PDAS values can be achieved by combining higher scanning velocity with lower laser power.

Laser technology has also played an important role in the field of composite material processing. Xu et al. [5] studied the carbon-fiber-reinforced composite (CFRP) cutting quality. Due to its properties of high specific strength, low density and excellent corrosion resistance, CFRP has been widely used in aerospace and automobile lightweight manufacturing as an important material. A nanosecond laser with a wavelength of 532 nm was applied to cut holes with a 2-mm-thick CFRP plate by using laser rotational cutting technology. The influence of different parameters on the heat-affected zone, the cutting surface roughness and the hole taper was explored, and the cutting process parameters were optimized. Using the optimized cutting parameters, the minimum value of the heat-affected zone, the cutting surface roughness and the hole taper could be obtained; they were 71.7 µm, 2.68 µm and 0.64°, respectively.

Silicon-carbide-fiber-reinforced silicon carbide ceramic matrix composite (CMC-SiC_f_/SiC) is a typical difficult-to-process material. Chen et al. [6] investigated the grooved morphology of CMC-SiC_f_/SiC using a dual-beam coupling nanosecond laser. Two kinds of scanning methods were set up according to the relationship between the spatial posture of the dual beams and the direction of the machining path. The CMC-SiC_f_/SiC grooving experiments were carried out along different feeding directions (transverse scanning and longitudinal scanning) by using a novel dual-beam coupling nanosecond laser. The results showed that the transverse scanning grooving section morphology had a V shape, and the longitudinal scanning groove section morphology had a W shape. The grooving surface depth and width of transverse scanning were larger and smaller than that of longitudinal scanning when the laser parameters were the same. The depth of the transverse grooving was greater than that of the longitudinal grooving when the laser beam was transverse and had longitudinal scanning, and the maximum grooving depth was approximately 145.39 µm when the laser energy density was 76.73 J/cm^2^. The thermal conductivity of the fiber had a significant effect on the local characteristics of the grooved morphology when using medium energy density grooving. The obvious recasting layer was produced after the laser was applied to CMC-SiC_f_/SiC when using high energy density laser grooving.

Water-jet-assisted laser processing technology can introduce new process advantages, such as reducing thermal effects. A novel coaxial-annulus-argon-assisted (CAAA) atmosphere was proposed to enhance the machining capacity of the water-jet-guided laser (WJGL) when dealing with hard-to-process materials in the study by Li et al. [7]. A theoretical model was developed to describe the two-phase flow of argon and the water jet. Simulations and experiments were conducted to analyze the influence of argon pressure on the working length of the WJGL beam, drainage circle size and extreme scribing depth on ceramic matrix composite (CMC) substrates. Single-point percussion drilling experiments were performed on a CMC substrate to evaluate the impact of machining parameters on hole morphology. On these bases, the CAAAWJGL was applied to scribe micro grooves on a CVD diamond, with a large depth-to-width ratio, good consistency and limited defects. The maximum depth-to-width ratio of the groove and depth-to-diameter ratio of the hole reached up to 41.2 and 40.7, respectively. The thorough holes produced by the CAAAWJGL demonstrated superior roundness and minimal thermal damage, such as fiber drawing and delamination. The average tensile strength and fatigue life of the CMCs specimens obtained through CAAAWJGL machining reached 212.6 MPa and 89,463.8 s, exhibiting a higher machining efficiency and better mechanical properties compared to femtosecond and picosecond laser machining. Moreover, groove arrays with a depth-to-width ratio of 11.5, good perpendicularity, and minimal defects on a CVD diamond were fabricated to highlight the feasibility of the proposed machining technology.

In addition, electric discharge machining is also a commonly used special machining method. Electrostatic field-induced electrolyte jet (E-Jet) electric discharge machining (EDM) is a newly developed micro-machining method. However, the strong coupling of the electrolyte jet liquid electrode and the electrostatic-induced energy prohibited it from being utilized in the conventional EDM process. Zhang et al. [8] proposed a method with two discharge devices connecting in serials to decouple pulse energy from the E-Jet EDM process. Through the automatic breakdown between the E-Jet tip and the auxiliary electrode in the first device, the pulsed discharge between the solid electrode and the solid workpiece in the second device can be generated. With this method, the induced charges on the E-Jet tip can indirectly regulate the discharge between the solid electrodes, giving a new pulse discharge energy generation method for traditional micro EDM. The pulsed variations in current and voltage generated during the discharge process in the conventional EDM process verified the feasibility of this decoupling approach. The influence of the distance between the jet tip and the electrode, as well as the gap between the solid electrode and the workpiece, on the pulsed energy demonstrates that the gap servo control method is applicable. Experiments with single points and grooves indicate the machining ability of this new energy generation method.

Introducing ultrasonic vibration in the manufacturing or material forming process is a very effective innovation. Li et al. [9] investigated the effect of ultrasonic vibration on the fluidity and microstructure of cast aluminum alloys (AlSi9 and AlSi18 alloys) with different solidification characteristics. Furthermore, the impact of ultrasonic vibration on the flow field during the molten metal filling process was analyzed using fluid simulation software (ANSYS-FLUENT). The results show that ultrasonic vibration can affect the fluidity of alloys in both solidification and hydrodynamics aspects. For the AlSi18 alloy without dendrite-growing solidification characteristics, the microstructure was almost not influenced by ultrasonic vibration, and the influence of ultrasonic vibration on its fluidity was mainly in hydrodynamics aspects. That is, appropriate ultrasonic vibration can improve fluidity by reducing the flow resistance of the melt, but when the vibration intensity is high enough to induce turbulence in the melt, the turbulence will increase the flow resistance greatly and decrease fluidity. However, for the AlSi9 alloy, which obviously has dendrite-growing solidification characteristics, ultrasonic vibration can influence solidification by breaking the growing (Al) dendrite, consequently refining the solidification microstructure. Ultrasonic vibration could then improve the fluidity of the AlSi9 alloy, not only from the hydrodynamics aspect but also by breaking the dendrite network in the mushy zone to decrease the flow resistance.

The ultrasonic-vibration-assisted pressing process can improve the fluidity and the uneven distribution of the density and particle size in the WC-Co powder. Chen et al. [10] used three-dimensional spherical models with the aid of the Python secondary development to simulate WC particles with a diameter of 5 µm and Co particles with a diameter of 1.2 µm. The forming process of the powder at the mesoscale was simulated by virtue of the finite element analysis software ABAQUS. The influence of the vibration amplitude on the fluidity, the filling density, and the stress distribution of WC-Co powder when the ultrasonic vibration was applied to the conventional pressing process was investigated. The simulation results show that the ultrasonic vibration amplitude had a great influence on the density of the compact. With an increase in the ultrasonic amplitude, the compact density also increased gradually, and the residual stress in the billet decreased after the compaction. From the experimental results, the size distribution of the billet was more uniform, the elastic after-effect was reduced, and the dimensional instability was improved.

Overall, the works published in this Special Issue show that high performance materials play an important role in the field of advanced manufacturing technology, and various energies such as laser, electric energy, ultrasonic vibration, and fluid energy can exert different technological advantages in the machining process. The introduction of new energy forms or the combination of various energy fields can significantly improve the processing effect for different materials, and this is one of the important development directions of future manufacturing technologies. The editors hope that the readers can discover the advantages of laser and energy field composite processing of high-performance material from the research results of this Special Issue, and gain some inspiration, which will promote the future research work.
